# Comparison of bag-valve-device ventilation and volume-controlled ventilation with laryngeal tube evo in intra-arrest ventilation—a prospective randomized study in human body donors

**DOI:** 10.1186/s44158-026-00458-9

**Published:** 2026-09-24

**Authors:** Cornelius Christopher Falk, Beate Brand-Saberi, Eckart Förster, Tamar Gelashvili, Harald Genzwürker, Jochen Hinkelbein, Annika Hoyer, Amy Thomasseck, Justin Trenkel, Jens Tiesmeier, Lydia Johnson Kolaparambil Varghese, Gerrit Jansen

**Affiliations:** 1 Ruhr University Bochum, Medical Faculty of Ruhr University Bochum, Universitätstraße 150, Bochum, 44801 Germany; 2 Institute of Anatomy, Ruhr University Bochum, Medical Faculty of Ruhr University Bochum, Universitätstraße 150, Bochum, 44801 Germany; 3 University Department of Anaesthesiology, Intensive Care Medicine, Emergency Medicine and Pain Medicine, Johannes Wesling Klinikum Minden, Ruhr University Bochum, Hans- Nolte-Straße 1, Minden, 32429 Germany; 4 Medical Faculty, University of Heidelberg, Im Neuenheimer Feld 672, Heidelberg, 69120 Germany; 5 Bielefeld University, Medical School OWL, Biostatistics and Medical Biometry, Universitätsstraße 25, Bielefeld, 33615 Germany; 6 Institute for Anaesthesiology, Intensive Care and Emergency Medicine, MKK-Hospital Luebbecke, Campus OWL, Ruhr-University Bochum, Virchowstraße 65, Lübbecke, 32312 Germany

**Keywords:** Resuscitation, Airway management, Ventilation, Artificial, Advanced cardiac life support

## Abstract

**Background:**

This study evaluated the effects of the new supraglottic airway device known as laryngeal tube evo on target parameters of ventilation therapy during mechanical chest compressions in intra-arrest ventilation with bag-valve-device ventilation vs. volume-controlled ventilation.

**Methods:**

Thiel-embalmed human body donors were included in a standardized resuscitation scenario. Ventilation was performed in a randomized order using the laryngeal tube evo for bag-valve-device ventilation and volume-controlled ventilation during mechanical cardiopulmonary resuscitation. Expiratory (Vt_*e*_) and expiratory tidal volumes per kg body weight (Vt_*e*_/kgBW); delta tidal volume (difference between ideal and expiratory tidal volume; ΔVt); leakage volume (*V*_leak_); peak (*P*_peak_); mean (*P*_mean_) and plateau (*P*_plat_) pressures and respiratory rates were recorded. The primary efficacy endpoint was Vt_*e*_.

**Results:**

Five body donors were included. Although one donor (20%) was successfully ventilated with all ventilation modes, three could only be ventilated with one of the modes (bag-valve-device ventilation = 20%; volume-controlled ventilation = 40%). One donor (20%) could not be sufficiently ventilated with any of the modes. Mean Vt_*e*_ was 91.73 ± 66.3 ml with bag-valve-device ventilation versus 142.93 ± 115.05 ml with volume-controlled ventilation. The comparison of both ventilation modes revealed higher Vt_*e*_ (50.27 ml; 95% confidence interval (CI): 7.56 to 92.97; *p* = 0.02), Vt_*e*_/kgBW (0.8 ml; 95% CI: 0.05 to 1.56; *p* = 0.04), *P*_mean_ (5.23 mbar; 95% CI: 4 to 6.47; *p* < 0.0001) and *P*_plat_ (10.36 mbar; 95% CI: 8.08 to 12.64; *p* < 0.0001), but lower ∆Vt (− 50.3 ml; 95% CI: − 93 to − 7.59; *p* = 0.02) and respiratory rates (− 10.83/min; 95% CI: − 17.29 to − 4.36; *p* = 0.001) for volume-controlled ventilation.

**Conclusions:**

Comparing volume-controlled ventilation with bag-valve-device ventilation for asynchronous ventilation during mechanical cardiopulmonary resuscitation using the laryngeal tube evo, volume-controlled ventilation resulted in higher Vt_*e*_ and Vt_*e*_/kgBW, but lower ∆Vt and respiratory rates. Future studies should evaluate laryngeal tube evo in prehospital use during mechanical cardiopulmonary resuscitation. When doubts regarding its effectiveness are present, synchronous ventilation (30:2) should be performed.

**Trial registration:**

German Clinical Trials Register; unique identifier: DRKS00037836. Registered on September 09, 2025 [1].

## Background

Since supraglottic airway devices (SGA) are easier to apply without interrupting chest compressions than performing tracheal intubation during cardiopulmonary resuscitation (CPR), the former are widely used worldwide, especially by non-physician emergency personnel [2, 3]. Use of SGA for airway management during cardiopulmonary resuscitation is recommended when rescuers achieve low intubation success rates [4]. In contrast to the guidelines of the American Heart Association, the current guidelines of the European Resuscitation Council (ERC) recommend the preferred use of an i-gel® laryngeal mask compared with a laryngeal tube for the first time due to favourable success and complication rates [4, 5]. Recently, an innovative new version of the laryngeal tube, the laryngeal tube evo (LT®evo) from VBM (VBM Medizintechnik GmbH, Sulz am Necker, Germany), has become available; however, the resuscitation effects of this device in the context of intra-arrest ventilation have not yet been evaluated.

Historically, intra-arrest ventilation has often been performed by using bag-valve-device ventilation in recent decades (particularly in Anglo-American countries) due to the lack of widespread availability of reliable emergency respirators [6–10]. In addition to bag-valve-device ventilation, the ERC now recommends the use of a mechanical ventilator with volume-controlled ventilation and a tidal volume of 6–8 ml/kg (predicted body weight), a maximum inspiratory oxygen fraction, a respiratory rate of 10/min, an inspiratory time of 1–2 s, a positive end expiratory pressure (PEEP) of 0–5 cmH2O, a peak pressure alarm at 60–70 cmH2O and a flow trigger in the off position [4]. To date, no studies have evaluated the effects of asynchronous intra-arrest ventilation with bag-valve-device ventilation compared to volume-controlled ventilation by using the novel LT®evo. Therefore, the effects of the LT®evo on the target parameters of ventilation therapy during bag-valve-device ventilation compared to volume-controlled ventilation during mechanical cardiopulmonary resuscitation (mCPR) were evaluated in a prospective human body donor study.

## Methods

After receiving approval from the ethics committee of Ruhr University Bochum in Ostwestfalen-Lippe, Bad Oeynhausen, Germany on September 9, 2025 (Ref. 2025-1341_2) and registration in the German Clinical Trials Register [1], this study involving human body donors was conducted in October 2025 at the Institute of Anatomy of Ruhr University Bochum in collaboration with “Klinisch-Anatomisches Forschungs- und Fortbildungszentrum”, Institute of Anatomy, Ruhr University Bochum, Germany [1]. The study utilized body donors who had been embalmed using the Thiel method, as this preservation technique best preserves the biomechanical properties of living lung tissue. Due to the limited availability of Thiel-embalmed body donors, in addition to the LT®evo various ventilation modes (such as chest-compression-synchronized ventilation with various chest-compression-synchronized ventilation pressures, bilevel positive airway pressure and continuous-positive-airway-pressure with tracheal tube and other SGA) were evaluated. To avoid interactions, both the order of the airway devices and the ventilation modes were randomized computer-assisted in advance and specified in a list for the studies. However, the data that were collected in this manner were analyzed prospectively and separately for each of the individual ventilation modes in this randomized order. This study was conducted in compliance with the ethical principles of the Declaration of Helsinki [11]. The structure of the paper is based on the current Consolidated Standards of Reporting Trials (CONSORT) guidelines [12].

### Inclusion and exclusion criteria

Adult Thiel-embalmed human body donors were included in this research [13]. Body donors with abnormal airways, tracheostomies, premortem adult respiratory distress syndrome and severe lung or thoracic injuries (such as undrained pneumothorax or severe aspiration) were excluded.

### General preparation of the human body donors

After an external post-mortem examination with documentation of the biological sex, actual body weight and size of the body donors was performed, the ideal body weight (IBW) was calculated on the basis of Broca’s formula [14]. This process was followed by tracheal intubation with Mallinckrodt tubes (male = 8.0 inner diameter in mm, female = 7.0 inner diameter in mm, cuff pressure = 40cmH2O) and bronchoscopy to verify correct tube position and remove secretions.

In preparation for the experiment, the lungs were recruited for four minutes by using pressure-controlled ventilation (inspiratory pressure (*P*_Insp_) = 35 mbar, PEEP = 12 mbar, respiratory rate = 10/min) via MEDUMAT Standard^2^ (WEINMANN Emergency, Medical Technology GmbH + Co., KG, Hamburg, Germany) and extubated after recruitment.

### Airway management

After lung recruitment, the LT®evo (VBM Medizintechnik GmbH, Sulz am Necker, Germany) was inserted according to predefined randomization. The size of the LT®evo was selected according to the manufacturer's specifications. The cuff pressure was limited to 40cmH_2_O and was checked via a cuff pressure device after insertion.

After each change in the airway device, the position was checked via bronchoscopy, and the lungs were again recruited by using five standardized ventilations via the MEDUTrigger (MEDUMAT Standard^2^, WEINMANN Emergency, Medical Technology GmbH + Co., KG, Hamburg, Germany) before ventilation was resumed. After four minutes of intra-arrest ventilation, the airway device was removed, and the next airway device was inserted according to the randomization list and checked once again as previously described.

### LT®evo

**Fig. 1:**
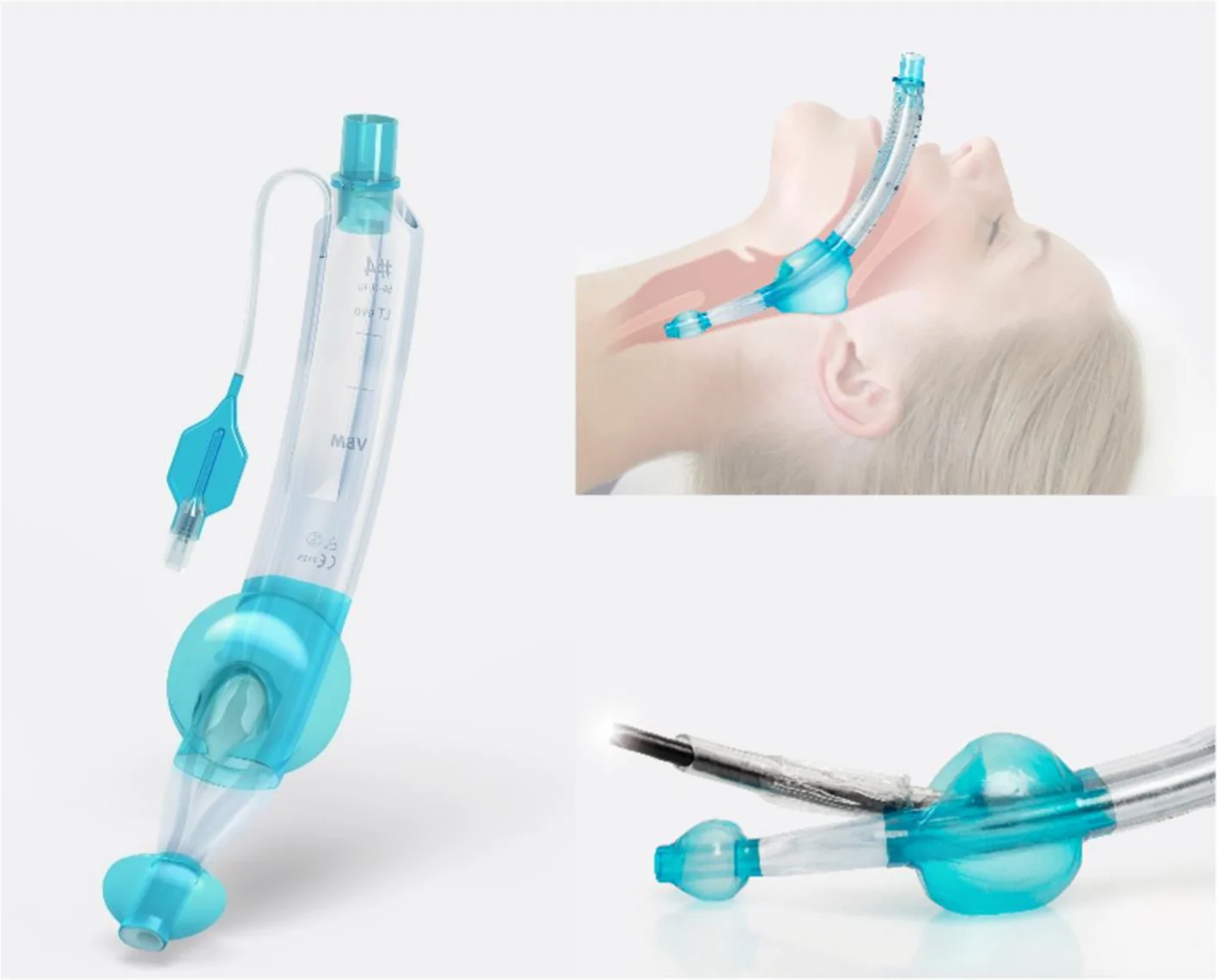
Illustration of the laryngeal tube evo

The LT®evo is a second-generation SGA and represents a complete redesign of the established laryngeal tube LTS-D. Specifically, this device is composed of more flexible and softer material than its predecessor and exhibits a modified shape that adapts to the anatomical contours of the relaxed upper airways. Moreover, its appearance is designed to ensure easy handling and to allow for easier insertion. Similar to its predecessor, the LT®evo has a proximal cuff to seal the oropharyngeal area with modified cuff geometry to enhance seal pressure (cuff pressure ≤ 60 cmH2O) and effective sealing, as well as a distal cuff to seal the oesophageal entrance. Additionally, its widened ventilation lumen and an integrated ramp at the end of the ventilation lumen facilitate fibreoptic-guided intubation through the device. An illustration of the LT®evo is presented in Fig. 1.

### Ventilation and chest compressions

Ventilation was performed with the assistance of MEDUVENT (WEINMANN Emergency, Medical Technology GmbH + Co., KG, Hamburg, Germany) for four minutes according to the predefined randomization. First, bag-valve-device ventilation was performed by specialists in anaesthesiology who also had years of experience in preclinical emergency medicine, with a target respiratory rate of 10/min being utilized. Second, volume-controlled ventilation (Vt_*e*_/kgBW = 7 mL/kgBW; respiratory rate = 10/min; PEEP = 3 mbar; maximum pressure limit (*P*_max_) = 40 mbar) for four minutes per airway device was utilized.

The chest compressions were performed in a standardized manner by using the mechanical chest compression device Corpuls-CPR (GS Elektromedizinische Geräte G. Stemple GmbH, Kaufering, Germany; frequency = 100/min; depth = 5.5 cm) [5]. Between the various resuscitation scenarios, the sonographic exclusion of pneumothorax was performed; if this condition was present, drainage was performed by inserting a chest drain in the Bülau position.

### Measurement of the ventilation parameters

**Fig. 2:**
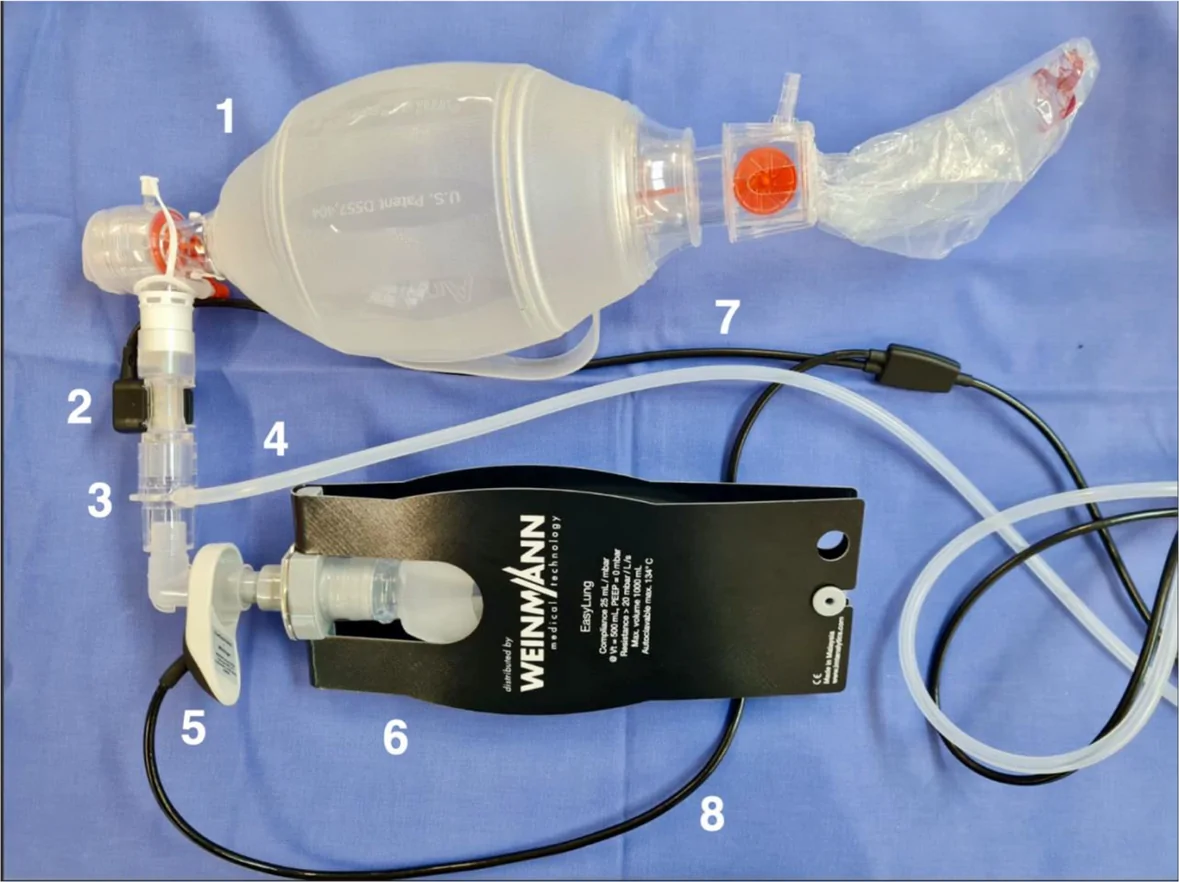
Measurements of airway pressure. Legend: 1, disposable resuscitator^1^; 2, FlowCheck sensor^2^; 3, connector for CO_2_ measuring tube^2^; 4, pressure measuring tube^2^; 5, MEDUtrigger^2^; 6, testing bag^2^ (as visualization of the connected device); 7, FlowCheck sensor connection line^2^; 8, MEDUtrigger connection line^2^; ^1^ Ambu Spur II, Ambu GmbH; ^2^ WEINMANN Emergency, Medical Technology GmbH + Co., KG

To measure and record the ventilation parameters for bag-valve-device ventilation during the experiments, the flow sensor of MEDUMAT Standard^2^ was modified (see Fig. 2). Therefore, parts of the measurement tubing system, which are used in mechanical ventilation to measure ventilation pressures, flow, and carbon dioxide (CO_2_) (including the FlowCheck sensor and adapter, CO_2_ measurement tube adapter, and pressure measurement tube), were used and installed between the bag valve and the airway device. The pressure measurement tube was connected to the CO_2_ adapter (see Fig. 2).

For volume-controlled ventilation, the MEDUVENT was used (WEINMANN Emergency, Medical Technology GmbH + Co., KG, Hamburg, Germany). The following parameters were recorded: Vt_*e*_ in ml, *V*_leak_ in percentage, *P*_peak_ in mbar, and *P*_mean_ in mbar. The calculated parameters included the ideal tidal volume (Vt_ideal_), Vt_*e*_/kgBW and ΔVt_*e*_ (Vt_ideal_−Vt_*e*_). A detailed overview of the recorded and calculated ventilation parameters is provided in Table 1. Sufficient ventilation was defined as the achievement of a Vt_*e*_ in excess of a dead space volume of 2 ml/kg body weight.

**Table 1:** Table of measured ventilation parameters and calculation methods

Parameters	Description	Calculation
Vt_*e*_ (ml)	Expiratory tidal volume, approximation for actual tidal volume	Sum of expiratory flow values (averaged over 5 breaths): Times for inspiration and expiration sought on pressure curve (pressure of approximately 0), transfer to flow curve, integral over expiration
Vt_*i*_ (ml)	Inspiratory tidal volume	Sum of the positive values from the beginning to the end of inspiration (end to beginning of expiration)
Vt_ideal_ (ml)	Calculated tidal volume based on the ideal body weight (IBW)	Calculated by the mechanical ventilator: IBW female (kg) = 45 + 2.3 × (height in cm/2.54 − 60) IBW male (kg) = 50 + 2.3 × (height in cm/2.54 − 60) multiplied by 6ml
∆Vt (ml)	Parameters for visualizing effective ventilation	∆Vt = Vt_ideal_−Vt_e_
MV_*e*_ (l/min)	Expiratory volume per minute	Minute average of the expiratory tidal volume measured by the device MVe = Vt × *F*, averaged over 5 breaths
*V*_leak_ (%)	Relative leakage volume	Integral of the flows over one minute (positive flows−negative flows) divided by the inspiratory minute volume ${V}_{\mathrm{l}\mathrm{e}\mathrm{a}\mathrm{k}}={\int}_{0}^{60}{f}_{+}\left(t\right)\mathrm{d}\mathrm{t}-|{\int}_{0}^{60}{f}_{-}\left(t\right)\mathrm{d}\mathrm{t}|$
*P*_peak_ (mbar)	Peak pressure	Mean value of the maximum airway pressure (peak pressure) per respiratory cycle over 1 min
*P*_mean_ (mbar)	Mean pressure	Average value of the pressure over one minute. All of the pressure values measured during a breath were totalled and divided by the number of time intervals in which the measurement was taken
*P*_plat_ (mbar)	Plateau pressure	Pressure value measured by the device in the last 30ms of inspiration, averaged over 5 breaths
*F*_manual_ (/min)	Manual ventilation frequency	Trend value measured by the device, averaged over 5 breaths

### Outcomes

The primary endpoint was Vt_*e*_. The secondary endpoints included ΔVt_*e*_, V_leak_, Vt_*e*_/kgBW, *P*_peak_, *P*_mean_, and *P*_Plat_.

### Statistical analysis

Data were first analysed descriptively according to the variable type, including means and standard deviations for continuous variables, as well as numbers and proportions for categorical variables. For the primary outcome, a linear regression model with a random intercept was used to account for potential intrabody-donor correlations. For all of the secondary outcomes, we once again used linear regression models with random intercepts. As effect measures, we report regression coefficients with 95% CIs and *p*-values (if appropriate). A *p*-value ≤ 0.05 was considered to indicate statistical significance. All analyses were performed using the statistical software SAS 9.4® (SAS Institute Inc., Cary, NC).

## Results

**Fig. 3:**
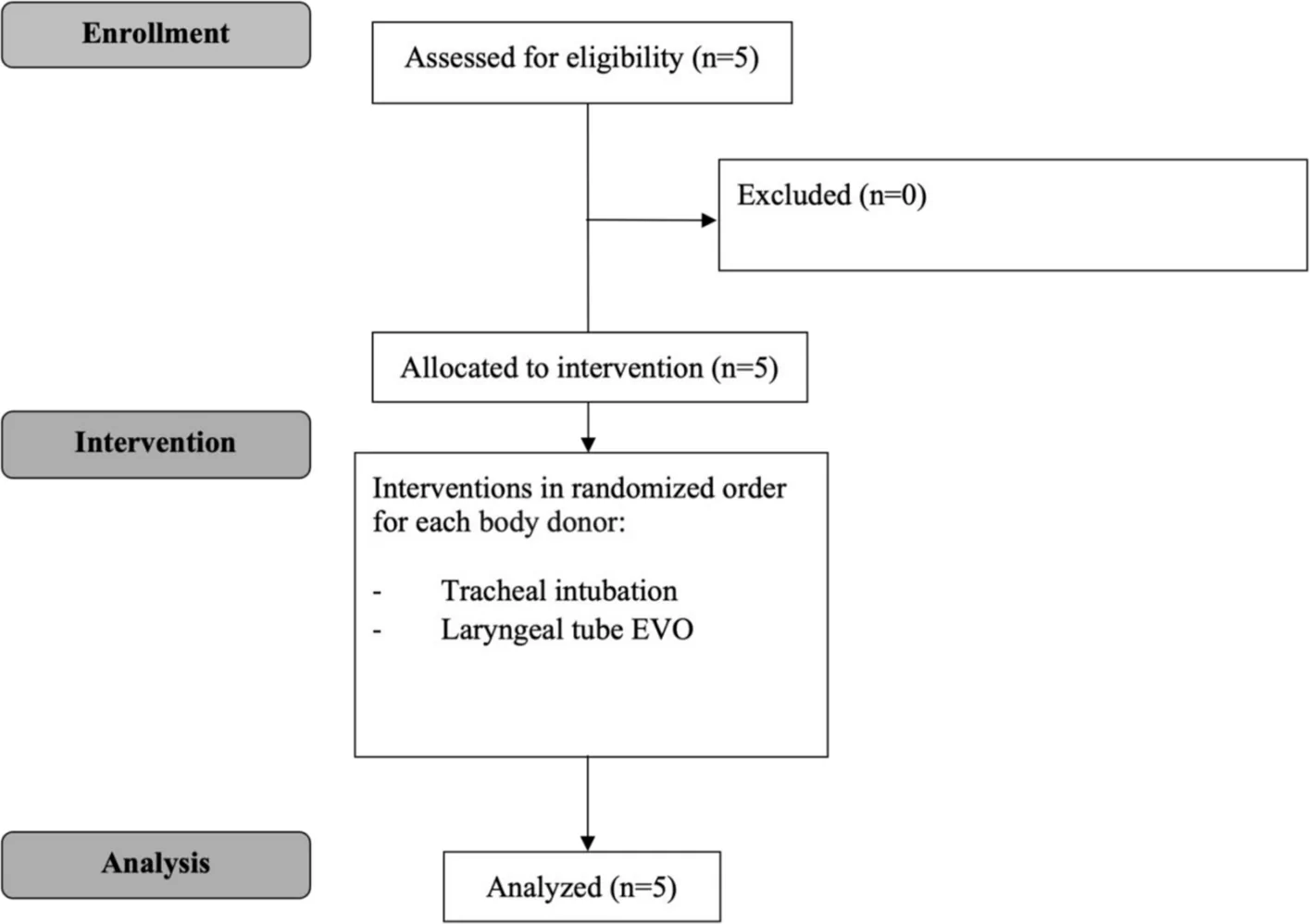
Flow diagram for the study protocol (based on CONSORT 2025)

Five human body donors were included after all of the inclusion and exclusion criteria were applied (see Fig. 3).

Table 2 presents the characteristics of the included body donors; Table 3 provides descriptive statistics of the ventilation parameters according to the different ventilation modes.

**Table 2:** Characteristics of the included human body donors

No	Sex (f/m)	Age (years)	Size (cm)	Body weight (kg)	Ideal body weight (kg)	Calculated alveolar dead space (2 ml/kg ideal body weight) (ml)	Ideal tidal volume (ml)	Body mass index (BMI)	Time-to-airway TI (sec)	Time-to-airway Laryngeal tube evo (Sec)	Pneumothorax during intervention	Comorbidities
1	m	79	172	65	72	144	504	22	10	7	None	Prostate carcinoma
2	f	86	152	50	52	104	364	22.5	30	2	Left	f-TEP, dementia
3	m	69	180	55	80	160	560	17	4	12	Right, left	None
4	f	90	162	70	62	124	434	26	8	2	None	Dementia, heart failure, diabetes mellitus, liver cirrhosis
5	f	86	165	57	65	130	455	21	8	13	None	Dementia, pancreatic carcinoma
Mean ± SD		82.0 ± 7.4	166.2 ± 9.4	59.4 ± 7.2	66.2 ± 9.4	132.4 ± 18.9	463.4 ± 66.0	21.7 ± 2.9	12.0 ± 2.9	5.75 ± 4.1

**Table 3:** Descriptive statistics of the measured parameters across the different ventilation modes

Variable	Overall (*n* = 66)	Bag-valve-device ventilation (*n* = 20)	Volume-controlled ventilation (*n* = 46)
Expiratory tidal volume (ml)			
Mean ± SD	127.42 ± 104.93	91.73 ± 66.31	142.93 ± 115.05
Median (Q1; Q3)	110.81 (42.63; 223.68)	52.29 (40.19; 145.89)	145.2 (42.77; 231.02)
Ideal tidal volume (ml)			
Mean ± SD	461.89 ± 67.15	463.4 ± 67.74	461.24 ± 67.63
Median (Q1; Q3)	455 (434; 504)	455 (434; 504)	455 (434; 504)
Δ Tidal volume (ideal – expiratory tidal volume) (ml)			
Mean ± SD	334.48 ± 130.86	334.48 ± 130.86	318.31 ± 133.29
Median (Q1; Q3)	336.17 (210.32; 470)	336.17 (210.32; 470)	309.89 (207.5; 437.23)
Inspiratory minute volume (l)			
Mean ± SD	4.69 ± 4.82	N/A^a^	4.69 ± 4.82
Median (Q1; Q3)	4.09 (3.04; 5.4)	N/A^a^	4.09 (3.04; 5.4)
Expiratory minute volume (l)			
Mean ± SD	1.37 ± 1.06	1.25 ± 0.89	1.42 ± 1.13
Median (Q1; Q3)	1.39 (0.38; 2.29)	1.33 (0.37; 2.03)	1.44 (0.43; 2.31)
Leakage volume (%)			
Mean ± SD	66.79 ± 24.76	68.93 ± 25.55	65.85 ± 24.64
Median (Q1; Q3)	68.86 (54.06; 87.97)	72.55 (58.82; 93.3)	66.17 (51.55; 87.67)
Tidal volume per kg body weight (ml)			
Mean ± SD	2.24 ± 1.89	1.66 ± 1.35	2.49 ± 2.05
Median (Q1; Q3)	1.94 (0.62; 3.4)	0.89 (0.62; 2.6)	2.86 (0.75; 3.52)
Peak pressure (mbar)			
Mean ± SD	23.01 ± 13.17	24.57 ± 16.4	22.33 ± 11.63
Median (Q1; Q3)	22.87 (12.09; 33.5)	27.85 (9.06; 35.51)	19.85 (12.09; 33.33)
Mean pressure (mbar)			
Mean ± SD	6.91 ± 3.76	3.27 ± 2.37	8.5 ± 3.1
Median (Q1; Q3)	6.44 (4.45; 9.98)	3.04 (1.08; 5.65)	7.69 (6.22; 11.83)
Plateau pressure (mbar)			
Mean ± SD	8.68 ± 7.92	1.5 ± 3.47	11.8 ± 7.26
Median (Q1; Q3)	7.2 (1.76; 13.34)	0.1 (0; 0.99)	8.03 (7.07; 16.67)
Respiratory rate per minute (/min)			
Mean ± SD	13.61 ± 15.33	21.22 ± 25.76	10.31 ± 4.72
Median (Q1; Q3)	10 (10; 10)	10 (8.76; 12.89)	10 (10; 10)

Legend: *SD* standard deviation, *Q* quartile, *N/A* not available

^a^MEDUMAT Standard^2^ does not measure the inspiratory minute volume

Table 4 shows the results of the linear mixed regression analyses with random intercepts of various ventilation parameters between volume-controlled ventilation and bag-valve-device ventilation. Comparisons of volume-controlled ventilation vs. bag-valve-device ventilation revealed higher Vt_*e*_ (50.27 ml; 95% CI: 7.56 to 92.97; *p* = 0.02), Vt_*e*_/kgBW (0.8 ml/kgBW; 95% CI: 0.05 to 1.56; *p* = 0.04), *P*_mean_ (5.23 mbar; 95% CI: 4.0 to 6.47; *p* < 0.001) and *P*_plat_ (10.36 mbar; 95% CI: 8.08 to 12.64; *p* < 0.001) but lower ∆Vt (− 50.3 ml; 95% CI: − 93 to − 7.59; *p* = 0.02) and respiratory rates (− 10.83/min; 95% CI: − 17.29 to − 4.36; *p* = 0.001) for volume-controlled ventilation.

**Table 4:** Results of linear mixed regression with random intercept for comparison of volume-controlled ventilation (reference) and bag-valve-device ventilation

Covariate	Mean difference (regression coefficient)	95% CI	*p*-value
Expiratory tidal volume (ml)	50.27	7.56 to 92.97	0.02
∆ tidal volume (ml)	− 50.3	− 93.0 to − 7.59	0.02
Leakage volume (%)	− 2.86	− 13.88 to 8.16	0.61
Tidal volume per kg body weight (ml/kg)	0.8	0.05 to 1.56	0.04
Maximum peak pressure (mbar)	− 2.19	− 8.07 to 3.7	0.46
Mean pressure (mbar)	5.23	4 to 6.47	< 0.001
Plateau pressure (mbar)	10.36	8.08 to 12.64	< 0.001
Respiratory rate (/min)	− 10.83	− 17.29 to − 4.36	0.001

The descriptive statistics of the measured parameters according to the different ventilation modes that were used for each of the body donors are provided in Table 5. Although one donor (20%; body donor 2) was successfully ventilated and achieved a Vt_*e*_/kgBW > 2 ml/kg with all of the ventilation modes, only three donors could be ventilated with one of the modes (bag-valve-device ventilation = 20% (body donor 5); volume-controlled ventilation = 40% (body donors 3 and 4)). Furthermore, one donor (20%; body donor 1) could not be sufficiently ventilated with any of the evaluated modes.

**Table 5:** Descriptive statistics of the measured parameters across the ventilation modes for each body donor

Variable	Bag-valve-device ventilation	Volume-controlled ventilation
Patient 1	*n* = 4	*n* = 9
Expiratory tidal volume ± SD (ml)	29.15 ± 9.08	3.55 ± 3.78
Delta tidal volume ± SD (ml)	474.86 ± 9.08	500.45 ± 3.78
Inspiratory minute volume ± SD (l)	N/A^a^	5.32 ± 0.68
Expiratory minute volume ± SD (l)	0.2 ± 0.13	0.04 ± 0.04
Leakage volume ± SD (%)	88.54 ± 16.11	96.35 ± 9.32
Tidal volume per kg body weight ± SD (ml)	0.45 ± 0.14	0.05 ± 0.06
Peak pressure ± SD (mbar)	17.8 ± 12.84	10.09 ± 1.71
Mean pressure ± SD (mbar)	2.25 ± 1.5	5.91 ± 0.57
Plateau pressure ± SD (mbar)	0.13 ± 0.13	7.4 ± 0.54
Respiratory rate ± SD (/min)	6.69 ± 2.71	9.69 ± 0.94
Patient 2	n = 4	n = 10
Expiratory tidal volume ± SD (ml)	181.04 ± 53.17	171.5 ± 61.68
Delta tidal volume ± SD (ml)	182.96 ± 53.17	192.5 ± 61.68
Inspiratory minute volume ± SD (l)	N/A^a^	2.47 ± 0.84
Expiratory minute volume ± SD (l)	1.87 ± 0.51	1.74 ± 0.62
Leakage volume ± SD (%)	66.43 ± 9.26	50.91 ± 21.14
Tidal volume per kg body weight ± SD (ml)	3.62 ± 1.06	3.43 ± 1.23
Peak pressure ± SD (mbar)	27.29 ± 18.24	17.2 ± 5.4
Mean pressure ± SD (mbar)	4.46 ± 2.98	7.53 ± 2.02
Plateau pressure ± SD (mbar)	1.26 ± 1	7.52 ± 2.58
Respiratory rate ± SD (/min)	9.66 ± 0.68	9.22 ± 2.48
Patient 3	n = 4	n = 9
Expiratory tidal volume ± SD (ml)	66.47 ± 45.72	251.15 ± 137.1
Delta tidal volume ± SD (ml)	493.53 ± 45.72	308.85 ± 137.1
Inspiratory minute volume ± SD (l)	N/A^a^	8.97 ± 9.8
Expiratory minute volume ± SD (l)	2.36 ± 0.18	2.46 ± 1.34
Leakage volume ± SD (%)	27.94 ± 18.67	63.81 ± 17.08
Tidal volume per kg body weight ± SD (ml)	1.21 ± 0.83	4.57 ± 2.49
Peak pressure ± SD (mbar)	20.54 ± 13.69	18.41 ± 6.17
Mean pressure ± SD (mbar)	1.73 ± 1.16	6.57 ± 1.8
Plateau pressure ± SD (mbar)	0.47 ± 0.43	6.47 ± 1.67
Respiratory rate ± SD (/min)	68.62 ± 20.72	14.3 ± 9.09
Patient 4	n = 4	n = 9
Expiratory tidal volume ± SD (ml)	46.54 ± 3.13	215.99 ± 42.58
Delta tidal volume ± SD (ml)	387.46 ± 3.13	218.01 ± 42.58
Inspiratory minute volume ± SD (l)	N/A^a^	3.73 ± 0.96
Expiratory minute volume ± SD (l)	0.4 ± 0.05	2.15 ± 0.43
Leakage volume ± SD (%)	88.26 ± 10.82	50.99 ± 10.58
Tidal volume per kg body weight ± SD (ml)	0.66 ± 0.04	3.09 ± 0.61
Peak pressure ± SD (mbar)	22.14 ± 14.83	32.38 ± 5.92
Mean pressure ± SD (mbar)	3.33 ± 2.22	11.43 ± 1.99
Plateau pressure ± SD (mbar)	0 ± 0.0	15.79 ± 3.15
Respiratory rate ± SD (/min)	9.65 ± 1.63	9.52 ± 1.44
Patient 5	n = 4	n = 9
Expiratory tidal volume ± SD (ml)	135.46 ± 29.21	69.3 ± 37.33
Delta tidal volume ± SD (ml)	319.54 ± 29.21	385.7 ± 37.33
Inspiratory minute volume ± SD (l)	N/A^a^	3.19 ± 1.42
Expiratory minute volume ± SD (l)	1.43 ± 0.25	0.7 ± 0.34
Leakage volume ± SD (%)	73.51 ± 0.0	68.87 ± 28.87
Tidal volume per kg body weight ± SD (ml)	2.38 ± 0.51	1.22 ± 0.65
Peak pressure ± SD (mbar)	35.08 ± 23.49	34.13 ± 12.75
Mean pressure ± SD (mbar)	4.58 ± 3.08	11.15 ± 3.4
Plateau pressure ± SD (mbar)	5.67 ± 6.72	22.28 ± 7.63
Respiratory rate ± SD (/min)	11.47 ± 1.65	8.94 ± 3.17

Legend: *SD* standard deviation, *N/A* not available

^a^MEDUMAT Standard^2^ does not measure the inspiratory minute volume

## Discussion

### Main findings

This study investigated the influence of the new LT®evo during intra-arrest ventilation on the target ventilation parameters of human body donors by comparing volume-controlled ventilation and bag-valve-device ventilation. A comparison of volume-controlled ventilation vs. bag-valve-device ventilation revealed higher Vt_*e*_, Vt_*e*_/kgBW, *P*_mean_, and *P*_plat_ but lower ∆Vt and respiratory rates for volume-controlled ventilation.

### Airway management in out-of-hospital cardiac arrest

Optimal airway management during CPR has been the subject of research worldwide for years. Specifically, due to the relative ease of learning, simple maintenance of skills, and quick placement without interrupting chest compressions, SGA are frequently used by nonphysician emergency personnel [4, 5]. The new LT®evo is an advanced laryngeal tube; due to its recent introduction, there is currently no evidence regarding its use and effects on intra-arrest ventilation during CPR. The present study (in which the insertion of this novel laryngeal tube was successful in all cases) is the first to evaluate the use of the LT®evo in the context of mCPR and its effects on intra-arrest ventilation while following the ventilation methods recommended in the guidelines, based on the use of a human body donor model [4, 5].

### Intra-arrest ventilation

Due to the fact that compression-only CPR is often recommended and performed by lay rescuers, intra-arrest ventilation is urgently required when resuscitation measures are continued by emergency medical services for re-oxygenation and CO_2_ elimination [4]. In contrast to other advanced cardiovascular life support measures, such as early defibrillation, there is currently much less evidence regarding the effects of different airway devices on intra-arrest ventilation. However, when oxygenation and ventilation are inadequate when advanced cardiovascular life support is provided, this is associated with poor outcomes [4, 8]. In view of the fact that no monitoring methods or only monitoring methods with significant limitations are available in the context of prehospital CPR, studies such as the present one are important for obtaining a better understanding of various variables.

Due to its widespread availability and ease of use in asynchronous ventilation, bag-valve-device ventilation is frequently used in intra-arrest ventilation, especially in the initial phase of CPR after the insertion of an SGA [6, 15]. In previous studies, it has been demonstrated to be noninferior to mechanical ventilation in terms of the rate of return of spontaneous circulation; therefore, it is considered to be an important backup option in the current ERC guidelines in the event of mechanical ventilation failure [4]. Conversely, the efficacy and safety of the procedure are strongly dependent on the user, as validated monitoring of the applied ventilation volumes, pressures, frequencies, and gastric insufflation for intra-arrest ventilation is rarely available as a standard measure for bag-valve-device ventilation. Moreover, capnography is not always available and/or reliable. Therefore, previous studies have emphasized the high prevalence of undetected hyperventilation and hypoventilation, as well as their adverse effects [6, 16–18]. This was also observed in the present study, even though the physicians performing bag-valve-device ventilation were anaesthesiologists experienced in emergency medicine. Although bag-valve-device ventilation using LT®evo appeared to be fundamentally possible in the present study, it was not possible to ensure sufficient ventilation exceeding the dead space volume and to achieve the targeted tidal volume in each body donor (Tables 3 and 4).

In contrast, mechanical intra-arrest ventilation exhibits several theoretical advantages that make its use appear to be sensible. First, the use of the emergency ventilator relieves the emergency team by providing an assistant who is now available for other tasks. Second, unlike bag-valve-device ventilation, the capnography that is available on many emergency ventilators allows for the detection of the correct tube position, the monitoring of resuscitation quality, and the detection of a return of spontaneous circulation, along with providing information on the prognosis during CPR [4, 19–22]. Finally, particularly with respect to volume-controlled ventilation, which is used frequently in intensive care medicine, it seems at least theoretically possible to safely apply the preselected tidal volume.

The available data indicate that, regardless of the type of ventilation used when employing an SGA such as the LT®evo, there are sometimes significant interindividual differences in the ventilation capability of different body donors during mCPR, as has also been observed in other studies [6, 23]. This scenario may explain the poorer blood gas values observed in patients receiving SGA during prolonged mCPR in clinical studies [6, 24].

Due to chest compressions, pressure gradients and reverse flow phenomena occur during both inspiration and expiration, which exert negative effects on the applied tidal volume. Consequently, it remains unclear how much of the applied tidal volume is actually available for alveolar ventilation [25]. In addition, particularly for SGA, pronounced leaks occur during mCPR, as was also observed in the present study for the new LT®evo [6, 23].

There are several possible causes for this aforementioned effect. First, dynamic head movements triggered by chest compressions and/or fixation-related changes in the airway anatomy of the body donors could have led to positional changes of the LT®evo during CPR, which could have reduced its tightness. Second, in contrast to bag-valve-device ventilation, a PEEP value of 3 mbar was selected for volume-controlled ventilation, which may have reduced the tightness of the LT®evo during mechanical ventilation. Finally, with respect to device-associated features, it is possible that the uniform cuff pressure of 40cmH_2_O that was utilized in the study was too low and that the blocking of the LT®evo to the manufacturer's recommended maximum value of 60cmH_2_O would have contributed to a further reduction in leakage.

The available data indicate that when intra-arrest ventilation is performed by using the new LT®evo, substantial interindividual differences are observed in terms of effective ventilation above the dead space volume (both with bag-valve-device ventilation and with volume-controlled ventilation). As long as prehospital monitoring methods that have been validated for the real-time monitoring of intra-arrest ventilation are not available, our findings support guideline recommendations of switching to synchronous ventilation at a ratio of 30:2 while using SGA (especially during mCPR) when there is clinical suspicion or uncertainty regarding relevant leaks or adequate ventilation until definitive airway management is achieved [4, 6].

### Limitations

This study has several limitations that must be considered when interpreting the results. First, a resuscitation and ventilation time of 76 min per body donor may influence the condition and tissue properties. This duration results from the use of five airway devices, with each device involving 3–4 ventilation modes per body donor. The multilateral use of body donors is due to the lack of Thiel-fixed body donors. Additionally, similarly long resuscitation and ventilation times can also be achieved in emergency medicine. Furthermore, the possibility that the various airway devices may damage the airways during insertion and use, thereby causing a scenario such as a pneumothorax. This potential effect should also have been reduced by randomization. In addition, lung damage (such as pneumothorax) also regularly occurs outside of the study. In this study, no association was found between the occurrence of pneumothoraces and the different forms of ventilation or the airway devices used. Due to the small number of participants, no conclusions can be drawn from this. Second, it can be assumed that the measurement results differ between Thiel-fixed cadavers and real patients, as tissue structure and elasticity may differ. Nevertheless, Thiel fixation is currently among the most appropriate methods used to preserve natural tissue characteristics during conservation. It is also a standard technique used for surgical training; therefore, it provides insightful impressions of intra-arrest ventilation with the LT®evo. In addition, laboratory conditions increase comparability by eliminating uncontrollable factors. Third, a P_max_ of 40 mbar was predefined for mechanical ventilation in the present study. As the study was designed and registered prior to the publication of the 2025 ERC Guidelines, the updated recommendations regarding ventilator settings during CPR could not be taken into account. To ensure comparability across the different airway devices investigated, the same P_max_ was applied to all devices. For the same reason, the cuff pressure for all airway devices was set at 40cmH_2_O, even though the maximum cuff pressure of 60cmH_2_O specified by the manufacturer for the LT®evo could affect the seal. Fourth, the limited number of test subjects (5 body donors) must be considered, as this fact may limit the representativeness of the results. Overall, despite its limitations, the study offers groundbreaking insights into the use of LT®evo under resuscitation conditions.

## Conclusions

Compared with bag-valve-device ventilation, asynchronous intra-arrest ventilation with volume-controlled ventilation using the LT®evo during mCPR resulted in higher Vt_*e*_ and Vt_*e*_/kgBW and lower ∆Vt and respiratory rates for volume-controlled ventilation.

Unlike volume-controlled ventilation, bag-valve-device ventilation may not provide a backup option to ensure adequate ventilation. In cases involving any doubts regarding the effectiveness of ventilation, synchronous ventilation (30:2) should be performed. Future studies should evaluate the role of the LT®evo for prehospital use and during mCPR.

## Trial registration

German Clinical Trials Register; unique identifier: DRKS00037836; registration date: September 09, 2025 [1].

## Data Availability

The datasets that were used and/or analysed during the current study are available from the corresponding author upon reasonable request.

## Abbreviations

LT®evo: Laryngeal tube evo
Vt_*e*_: Expiratory tidal volume
CI: Confidence interval
Vt_*e*_/kgBW: Vt_*e*_ per kilogram of body weight
∆Vt: Difference between Vt_ideal_ and Vt_*e*_ (Vt_ideal_−Vt_*e*_)
*V*_leak_: Leakage volume
*P*_peak_: Peak pressure
*P*_mean_: Mean pressure
*P*_plat_: Plateau pressure
mCPR: Mechanical cardiopulmonary resuscitation
SGA: Supraglottic airway device
CPR: Cardiopulmonary resuscitation
ERC: European Resuscitation Council
PEEP: Positive end-expiratory pressure
CONSORT: Consolidated Standards of Reporting Trials
IBW: Ideal body weight
*P*_Insp_: Inspiratory pressure
*P*_max_: Maximum pressure
CO_2_: Carbon dioxide
Vt_ideal_: Ideal tidal volume
